# Impact of Jiggling Exercise as Conservative Treatment for Hip Osteoarthritis: A Report of Two Cases

**DOI:** 10.1155/2020/2804193

**Published:** 2020-05-04

**Authors:** Yohei Teramoto, Kensuke Fukushima, Tomohisa Koyama, Yoshihisa Ohashi, Katsufumi Uchiyama, Naonobu Takahira, Masashi Takaso

**Affiliations:** ^1^Department of Orthopaedic Surgery, Kitasato University School of Medicine, 1-15-1 Kitasato, Minami-ku, Sagamihara, Kanagawa 252-0374, Japan; ^2^Department of Rehabilitation, Kitasato University School of Allied Health Sciences, Japan

## Abstract

Total hip arthroplasty has been recognized as a feasible treatment for hip osteoarthritis, especially in advanced and terminal stages, but whether it is the best treatment for patients who are younger, have comorbidities, and/or are likely to have low compliance to medical treatment is unknown. Jiggling exercise, which involves the continuous shaking of the foot and leg in small steps, has been reported to be the easiest and a less invasive exercise for patients with hip osteoarthritis. We herein report two patients who performed jiggling exercise as conservative treatment and had successful outcomes. The first case was a 28-year-old woman with advanced-stage hip osteoarthritis that could not be treated with hip preservation surgery; furthermore, the patient refused to undergo total hip arthroplasty because of her young age. The second case was a 74-year-old woman with terminal-stage hip osteoarthritis in whom total hip arthroplasty was deemed not feasible because of possible low compliance due to mental disorder. One year after the initiation of the jiggling exercise, both patients had remarkable clinical improvement. Three years later, remarkable joint remodelling was also revealed in plain radiographs. Jiggling exercise might be a feasible conservative treatment option for joint preservation.

## 1. Introduction

Hip disorders such as hip osteoarthritis (OA) directly affect patients' activities of daily living (ADLs). Advanced- and terminal-stage hip OA causes severe restriction of hip range of motion (ROM) and hip pain. Total hip arthroplasty (THA) has been recognized as a feasible treatment for hip OA especially in advanced and terminal stages. THA has a great advantage in terms of early rehabilitation and recovery of ADL [1] as well as successful longevity [2, 3]. However, disadvantages and complications after THA, such as lack of durability and dislocation, have also been reported [4]. In addition, the question remains whether THA is the best treatment solution for patients who are younger, have comorbidities, and/or are likely to have low compliance to medical treatment [5].

**Figure 1:**
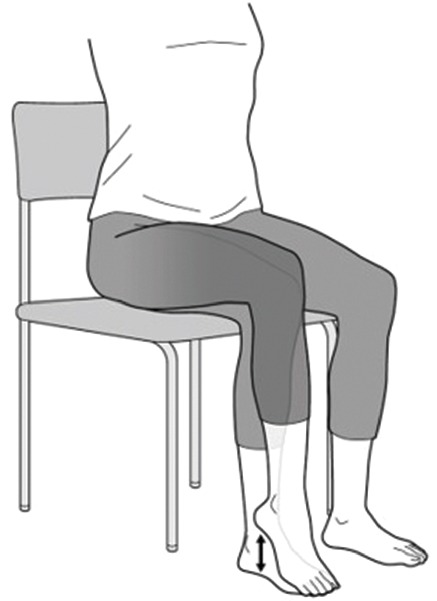
Jiggling exercise method. The patient continuously shakes his or her foot and leg in small steps whilst sitting on a chair for at least 30 minutes a day.

Jiggling exercise, which involves the continuous shaking of the foot and leg in small steps (Figure 1), has been reported to be the easiest and a less invasive exercise for patients with advanced- and terminal-stage hip OA [6]. Some case reports describing the efficacy of jiggling exercise for hip OA based on clinical and radiographic results have been published only in Japanese [7–9].

We have attempted jiggling exercise as conservative treatment for patients with advanced- and terminal-stage hip OA and patients who note OA progression after performing joint preservation surgery such as hip osteotomy and hip arthroscopy. We believe that patients who refuse to undergo THA or have contraindication(s) to THA (e.g., relatively young age, presence of comorbidities, and low compliance to medical treatments) might be good candidates for jiggling exercise. Patients are instructed to shake their foot and legs continuously in small steps whilst sitting on a chair for at least 30 minutes a day and for two sets, or for as long as the patients can tolerate the exercise. We also coadminister medications such as nonsteroidal anti-inflammatory drugs (NSAIDs) to the patients especially during the initial application of the exercise.

Our aim is to report typical cases of our two patients who were successfully treated with jiggling exercise. Both patients provided written consent for the publication of the case reports, including patient information and accompanying images.

## 2. Case Presentation

### 2.1. Case 1

**Figure 2:**
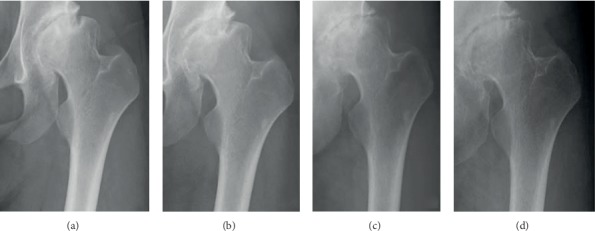
Plain radiograph for case 1, showing the hips at (a) first admission and at (b) 1 year, (c) 3 years, and (d) 4 years after the initiation of jiggling exercise.

A 28-year-old woman complained of severe left hip pain and difficulty walking. The pain started to develop when she was in her early twenties, with the intensity increasing with the intensity of physical work. She consulted a nearby orthopaedic department. The attending physician diagnosed her as having hip OA and referred her to our hospital for advanced examination and treatment. The patient was a nurse. She had no clinical history of treatment for hip disorders such as developmental dysplasia of the hip in childhood. With regard to her hip pain, she reported that the pain was present both at rest and during walking. Regarding her left hip, she additionally had tenderness at the femoral triangle of Scarpa, and both Patrick's test and anterior impingement test had positive results. The ROMs of her left hip were 130° in flexion, 20° in abduction, 30° in adduction, 30° in external rotation, and 10° in internal rotation, whereas ROMs of her right hip were limited especially in abduction and internal rotation, and she reported groin pain at the end of the motion. Her Japanese Orthopaedic Association (JOA) hip score was 57/100. Her left hip radiograph showed degenerative arthritis categorised as Tönnis grade 2, and bone cysts were noted on the femoral heads (Figure 2(a)).

Since improvements of joint congruity and space width were not investigated in hip abduction and adduction radiographs, we concluded that joint preservation surgeries such as periacetabular osteotomy could not be indicated for this patient. Moreover, she refused to undergo THA because of her young age. She was treated with NSAIDs and was instructed to perform general muscle and ROM exercises around the hip joint for 6 months. However, her hip pain did not improve, and repeat radiographs revealed OA progression. Hence, we recommended intensive jiggling exercise combined with medication.

One year after the initiation of jiggling exercise, the patient's hip pain notably improved, despite the absence of changes in the hip radiographs (Figure 2(b)). She was able to interrupt NSAID treatment. Three years later, the patient's hip pain remarkably improved and the radiographs of the hip joint showed notable improvement in terms of congruity and space width (Figure 2(c)). At the last follow-up, 4 years after the indication of the jiggling exercise, radiography revealed improved joint remodelling (Figure 2(d)). The ROMs of her left hip were slightly increased as 130° in flexion, 30° in abduction, 30° in adduction, 40° in external rotation, and 10° in internal rotation. Additionally, the JOA hip score was 84/100, which was assessed to be clinically effective.

### 2.2. Case 2

A 74-year-old woman was referred to our department for examination and treatment of severe left hip pain. The patient underwent surgical intervention for breast cancer 10 years before. In addition, she has been treated for schizophrenia for more than 30 years, and her communication skills were hindered by her mental disorder. Although she could perform her main ADLs whilst using a wheelchair, she complained of severe hip pain on sitting and when transferring to a wheelchair and thus required a high level of assistance.

**Figure 3:**
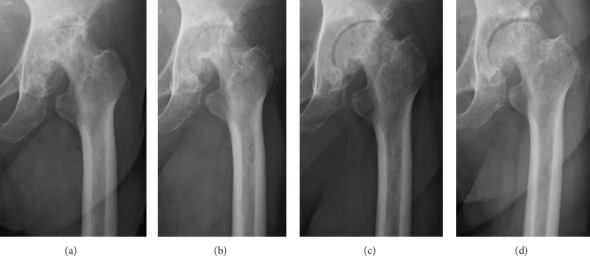
Plain radiograph for case 2, showing the hips at (a) first admission and at (b) 1 year, (c) 2 years, and (d) 3 years after the initiation of jiggling exercise.

She presented with tenderness at the femoral triangle of Scarpa on her left hip, and both the Patrick's test and anterior impingement test had positive results. The ROMs of her left hip were 60° in flexion, -20° in extension, 10° in abduction, 10° in adduction, 20° in external rotation, and 10° in internal rotation, which were severely limited. The radiograph on her left hip showed degenerative arthritis categorised as Tönnis grade 3, terminal OA (Figure 3(a)).

Considering her comorbidities and ADLs, THA was concluded to be too invasive for the patient. Since she could follow simple instructions, we prescribed an intensive jiggling exercise without additional medication. One year after the initiation of jiggling exercise, the patient's hip pain and ROM remarkably improved, and the plain radiograph of the hip revealed some improvement of joint congruity (Figure 3(b)). After 2 years, she could walk indoors with a cane. The radiographs of the hip joint showed remarkable improvement of joint congruity and space width (Figure 3(c)). After 3 years, at the last follow-up, her pain had already disappeared. The ROMs of her left hip were 120° in flexion, 40° in abduction, 30° in adduction, 50° in external rotation, and 20° in internal rotation, which were remarkably improved. Furthermore, plain radiographs revealed improving joint remodelling (Figure 3(d)).

## 3. Discussion

Conservative treatment for patients with advanced- and terminal-stage hip OA and patients with OA progression after joint preservation surgery is quite limited. Many guidelines for OA management recommend low-impact exercise such as ROM/flexibility, quadriceps strengthening, and aerobic exercises (land or water based) [10]. Teirlinck et al. [11] reported the results of a multicentre, pragmatic randomised controlled trial for patients with hip OA who underwent physical therapy, and they observed a difference in function at the 3-month follow-up, but there was no difference at the 12-month follow-up. In addition, performing exercise for patients with advanced- and terminal-stage hip OA might increase hip joint pain.

With regard to treatment using medications, many guidelines consistently recommend acetaminophen as first-line pharmacologic management of OA [10]. However, acetaminophen treatment might not affect the pathological mechanism of OA. Thus, its effectiveness might be temporary for patients with advanced- and terminal-stage hip OA. NSAIDs are also generally used in the treatment of OA. The Coxib and Traditional NSAID Trialists' Collaboration [12] reported on the side effects of NSAIDs, including selective COX-2 inhibitors and traditional NSAIDs, based on the results of a meta-analysis and stated that all NSAID regimens increased upper gastrointestinal complications compared to placebo. Emkey et al. [13] reported that the rate of pain relief was significantly lower for participants treated with tramadol/acetaminophen in combination with COX-2 inhibitors compared with participants treated with placebo and a COX-2 NSAID. However, in this multicentre, placebo-controlled study intended for patients with OA, tramadol recipients frequently had central nervous system complications [14]. Since hip OA is a chronic and degenerative disorder, longitudinal administration of drugs might increase the risks of side effects. We believe that easy administration and increasing the dose of the medicine should be avoided as much as possible.

THA is advantageous in terms of early postoperative pain relief and rehabilitation for patients with advanced- and terminal-stage hip OA. However, there is a risk of complications such as aseptic loosening and/or dislocation especially in relatively younger, active, and nonadherent patients [15]. Furthermore, we should consider the management for patients who cannot undergo THA because of physical and/or social constraints or those who refuse to undergo THA.

Jiggling exercise was first described as a conservative treatment for patients with OA progression following hip osteotomy by Hiromatsu and Inoue [6]. The concept of the exercise was developed as a form of continuous passive motion (CPM) exercise, which was described by Salter et al. [16, 17] who investigated the biological effect of CPM on the healing of full-thickness articular cartilage using rabbit model. Many studies have followed the study of Salter et al., and CPM has been described to contribute to the improvement of joint health by preventing joint stiffness, preserving normal articular tissue with better histologic and biologic properties and improving the ROM, compared with joint immobilisation and intermittent active motion [18]. Since jiggling exercise was designed to be much simpler and easier than CPM, it could be more feasible for patients to perform and continue as home exercise. Although we did not assess the effectiveness of joint remodelling, we think that the medications for pain control should be combined to perform and continue the exercise.

In the present report, our two patients who performed jiggling exercise were observed radiographically to have remarkable joint remodelling. Similar remodelling has been noted after femoral valgus osteotomy for patients with advanced- and terminal-stage hip OA [19]. Furthermore, a study reported the effectiveness of muscle-release operation for patients with advanced- and terminal-stage hip OA [20]. Maniwa et al. [9] have investigated the tension of the muscle around the hip joint before and after jiggling exercise. They reported that the tension of the muscle around the hip joint in patients with hip OA significantly decreased after performing jiggling exercise. Changes in mechanical environment of the joint might induce the body's inherent self-repair ability and promote natural healing of the hip joint. We believe that jiggling exercise has effects not only on pain relief and improving range of motion but also on promoting natural healing and joint preservation of the hip joint.

We have indicated jiggling exercise mainly for patients who show advanced- and terminal-stage hip OA and cannot undergo THA for some reason. The cases that we have treated with jiggling exercise have been very few to date. In addition, to confirm the clinical effectiveness of jiggling exercise, a relatively longer observation time compared with other treatments might be needed. The effectiveness of jiggling exercise for the patients with early-stage OA and other hip disorders such as labral tear, femoroacetabular impingement (FAI), and osteonecrosis of femoral head (ONFH) remains unclear. Further case-control and prospective randomised control studies with a sufficient number of patients are needed to clarify the effectiveness and limitations of jiggling exercise.

## 4. Conclusion

We have described two typical cases of patients with advanced-stage hip OA who were successfully treated with jiggling exercises. Although radiographic joint remodelling took a relatively longer time, the two patients who performed jiggling exercise as conservative treatment showed remarkable clinical improvement. Jiggling exercise might be a feasible conservative treatment option for hip OA.
